# Supplementary material for: Non-destructive defect detection in powder metallurgy automotive oil pump stators using acoustic signals and machine learning classification
Source: PLoS One. 2025 Nov 17;20(11):e0334454. doi: 10.1371/journal.pone.0334454 (PMC12622811; doi:10.1371/journal.pone.0334454)
Supplement: Attachment — Submitted filename: ReviewResponse30041404.docx [file pone.0334454.s001.docx]

**Manuscript Title:**

**Non-destructive defect detection in powder metallurgy components using acoustic signals and machine learning classification**

**Manuscript PONE-D-25-28300R1**

**Dear Editor in Chief,**

Thank you for taking the time to evaluate the suitability of our paper for your journal. The suggestions offered by the reviewers have been quite helpful. We have incorporated the suggested edits and have highlighted them in the main text. In addition, we also answered the questions posed by the reviewers point by point in the following sheet. We sincerely thank the reviewers for their efforts and appreciate their help in improving the overall quality of the work.

Kind regards,

Abbas Rohani

**Additional Editor Comments:**

The manuscript must be revised based on the reviewers’ comments plus the following issues, 1) A separated file must be provided for the authors’ answers to the comments, one by one. Moreover, all changes must be yellow-colored highlighted sentences in the revised article. The track changes condition is not suggested.

**Answer:**  We sincerely appreciate your guidance. The manuscript has been carefully revised according to the reviewers’ comments. A separate file containing our point-by-point responses to all comments has been prepared. In addition, all modifications in the revised manuscript are highlighted in yellow, as requested.

2) No abbreviations should be used in the keywords. Moreover, they must be also found in the abstract or the title.

**Answer:**  The keywords have been revised to remove abbreviations, and they are now consistent with the terms used in the title and the abstract

3) The introduction is lengthy. Only 3 pages are enough. Moreover, the novelty of the manuscript must be highlighted in the introduction, compared to the literature review.

**Answer:**  The introduction has been shortened to within three pages, and the novelty of the manuscript has been clearly emphasized in comparison with the existing literature.

4) All formulations need references, unless they were extracted or introduced by the authors.

**Answer:**  All formulations in the manuscript have been properly referenced.

5) The scale bar must be provided for macroscopic and microscopic images, such as Fig. 1.

**Answer:**  The scale bar has now been added to all images, including Fig. 1. In addition, the outer diameter of the stators (5.00 ± 0.05 cm) has been specified in the figure caption to provide precise dimensional information

6) Generally, the discussion is poor. The obtained results must be described firstly and then; they must be compared to the other results of other articles. This technical issue could be also found from the low number of references.

**Answer:**  We sincerely appreciate your valuable comment. Following your suggestion, Table 10 has been added at the end of the manuscript to summarize recent studies in the field of fault diagnosis using imaging, acoustic analysis, and related approaches. This addition not only highlights the position of the present study within the broader body of research but also provides a clear comparison with previous works, thereby strengthening the depth and comprehensiveness of the discussion section.

7) The number of references must be extended to 35-40 references, at least, based on recent published articles in 2020-2025.

**Answer:**  Thank you for your recommendation. In line with your earlier suggestion, the number of up-to-date references relevant to the scope of this research has been increased, particularly by including studies published between 2020 and 2025.

8) “Conclusion” must be changed to “Conclusions”.

**Answer:**  The section title has been revised from “Conclusion” to “Conclusions” accordingly.

**Reviewer # 1**

I recommend the paper for major revision with the following comments:

**1:** The title of the paper is too general, especially since the authors mention “powder metallurgy components” without specifying the material. This approach overgeneralizes the results obtained from a limited number of samples to the entire manufacturing process.

**Answer:**  Thank you for your valuable comment. We agree with your observation that the previous title was too general. To address this concern and to more accurately reflect the specific scope of our study, we have revised the title to:

*“Non-destructive defect detection in powder metallurgy automotive oil pump stators using acoustic signals and machine learning classification.”*

This revision avoids overgeneralization and specifies the exact type of component investigated, ensuring that the results are clearly contextualized within automotive oil pump stators manufactured by powder metallurgy

2: You should avoid using overly polished or complicated text in a research paper. For example, the sentence “Furthermore, PM manufacturing typically involves high production volumes, necessitating not only accurate defect detection but also rapid inspection methods” is unnecessarily complex. It uses correlative conjunctions within a present participle clause, which makes it difficult to follow. Also, avoid combining phrases like “due to” and “however” in one sentence. It would be clearer to restructure the sentence using a single transition word such as “although.” Initially, I mentioned this issue in the abstract, but after reviewing the rest of the paper, I recommend checking the entire text. Although the language is grammatically correct, it is sometimes hard to read. A research paper should use clear, direct, and consistent structures — it should not include a variety of sentence forms like TOEFL or IELTS essays.

**Answer:**  We have carefully reviewed the entire manuscript and revised the text wherever possible to simplify complex sentences, improve clarity, and ensure direct and consistent expression throughout the paper.

3: The advantage of using machine learning (ML) in this research is not mentioned in the abstract. It should be explained how this method can be effective and how it compares with other conventional methods. Currently, the abstract only highlights the necessity of using acoustic signal testing, without emphasizing the role or benefits of ML. Additionally, instead of providing a long general introduction in the abstract, it would be more useful to include details about the algorithm training process. Overall, the abstract needs to be completely restructured.

**Answer:**  We sincerely thank the Reviewer for this valuable comment. In the revised version, the Abstract has been improved to explicitly highlight the advantages of machine learning over conventional threshold-based inspection techniques, emphasizing its ability to detect complex, non-linear acoustic signal patterns that are often missed by traditional approaches. In addition, the training process of the algorithms has been clarified by specifying that time- and frequency-domain features were extracted from the collected acoustic data and used to train and validate four supervised ML classifiers (SVM, k-NN, MLP, and RBF). Furthermore, the comparative results of these models have been briefly summarized to demonstrate their performance, with the RBF model achieving 100% accuracy.

4: How is it possible that SVM and MLP neural network are listed as keywords in your paper but are not mentioned in the title or abstract? These important terms should at least appear in the abstract to reflect the main methods used in the study.

**Answer:**  We appreciate the Reviewer’s insightful observation. In accordance with your suggestion and the Editor’s guidance, the list of keywords has been carefully revised, and the Abstract has been rewritten to ensure that all major methods, including SVM and MLP, are explicitly mentioned. This issue has been fully addressed in the revised manuscript, and the updated list of keywords can be reviewed for confirmation

5: Please avoid introducing abbreviations for the first time without proper definition. When using an abbreviation, it should be introduced once with its full form, and then only the abbreviation should be used throughout the paper. For example, you have introduced “PM” and “ML” several times in different sections. Please check and correct this issue for all abbreviations in the text.

**Answer:**  We sincerely thank the Reviewer for this valuable comment. Following your suggestion, we have carefully revised the manuscript to ensure that each abbreviation is introduced only once with its full form at the first appearance, and then consistently used throughout the text. Specifically, abbreviations such as “PM” and “ML” have been corrected accordingly. This revision has been applied uniformly across all sections of the manuscript.

6: In the introduction, several long sentences make it difficult to follow the ideas clearly. The sentence structures are similar to those typically found in TOEFL reading passages, which are unnecessarily complex for a research paper. I recommend simplifying and restructuring these sentences to improve clarity and readability.

**Answer:**  We appreciate the reviewer’s valuable comment. Following the editor’s guidance to reduce the introduction to a maximum of three pages, this section has been revised accordingly. In addition, the issue rightly noted by the reviewer regarding long and complex sentence structures has been carefully addressed, not only in the introduction but also throughout other sections of the manuscript to enhance clarity and readability.

7: The introduction is too long, and the ideas in the paragraphs are not well connected. As a result, it is difficult for the reader to follow the overall direction of the paper. Moreover, the extended explanation about different types of non-destructive inspection methods makes the introduction unnecessarily lengthy and somewhat boring. In particular, the second paragraph does not contribute to the main objective of the paper. Instead, it seems to list information without offering a clear link to the research focus. Therefore, I suggest reorganizing and shortening the introduction to improve clarity, relevance, and reader engagement.

**Answer:**  We sincerely appreciate your valuable feedback. In line with your recommendation and the editor’s suggestion, the introduction has been thoroughly revised and reorganized. The section was shortened to improve clarity, eliminate redundancies, and strengthen the logical flow of ideas. The part concerning non-destructive inspection methods was also streamlined to ensure relevance to the research objectives. We hope that these revisions adequately address your concern.

8: The long explanation of vibration non-destructive testing in paragraph 3 of the introduction may be boring for the reader. It would be better to shorten this section and focus more on information directly relevant to the study.

**Answer:**  We appreciate your observation. This section has been shortened and simplified to avoid unnecessary detail. The primary aim of including it was to introduce the subject and highlight the importance of applying machine learning methods in this research area. In line with your valuable feedback, we have revised and restructured this part to improve clarity and relevance.

9: The literature review in the introduction related to acoustic testing is insufficient. Instead of listing different types of quality control methods and inspections in paragraph 2 and providing a long introduction to vibration testing at the beginning of paragraph 3, please include more detailed information about how acoustic testing differs from other methods. Also, explain the necessity of using this method compared to others, recent developments in acoustic testing, and its advantages.

**Answer:**  We appreciate your comment regarding the coverage of acoustic testing in the introduction. The introduction has been thoroughly revised and streamlined to avoid unnecessary elaboration. However, based on our review and the available references, there is very limited research on defect detection in industrial components produced by powder metallurgy, particularly in the automotive sector. Therefore, to highlight the importance of acoustic testing, we referred to related studies where applicable, in order to clarify its relevance and advantages compared to other methods. Additionally, Table 10 has been added to the manuscript, providing a comprehensive overview of the literature in this area. As shown in the table, most existing studies focus on defect detection using image-based techniques, which further emphasizes the novelty and significance of our work in applying acoustic testing for industrial defect detection.

10: Before the last paragraph of the introduction, which presents the novelty of the paper, the authors should clearly elaborate on the research gap and the necessity of conducting this study. Specifically, the following questions need to be addressed in detail:

- What are the necessities of using machine learning classification in this context?

- What advantages does the proposed method offer over existing approaches?

I recently reviewed a paper for PLOS One that used classification for defect detection in aluminum samples. I am interested to know whether changing the materials, manufacturing methods, or machine learning algorithms can contribute to solving real-world problems. If this method provides new insights or tools for researchers, the authors should explain this clearly in the research gap section of their paper.

**Answer:** We appreciate the reviewer’s valuable comment. In accordance with your suggestion, the necessity of using machine learning classification in this context and the advantages of the proposed method compared to existing approaches have been clarified at the end of the introduction. In particular, the study by Han et al. [Han, Ying, Xingkun Li, Gongxiang Cui, Jie Song, Fengyu Zhou, and Yugang Wang. Multi-defect detection and classification for aluminum alloys with enhanced YOLOv8. PloS One, 20(3), e0316817, 2025] was reviewed. As also highlighted in our introduction, nearly all of these studies are image-based, which suffer from certain drawbacks such as being time-consuming, costly, and lacking sufficient speed for continuous production lines. These limitations provided the main motivation for our research, where we propose a machine learning approach based on acoustic signals that enables faster, cost-effective, and more practical defect detection for real-world applications.

11: At the end of the introduction, the authors should provide the reader with a clear summary of the novelty and methodology. This should include how the samples were prepared, how the algorithms were trained, and whether different algorithms were compared to find the best one. It should also explain how the accuracy of the models was evaluated, if train-test splitting was used to assess the ability to predict new samples, and what materials were tested. All these details should be clearly defined in the last paragraph of the introduction.

**Answer:**  In the final paragraph of the introduction, we highlighted the research gap and the novelty of the study, while briefly outlining the main steps of the methodology, including sample preparation, algorithm training, and model evaluation. However, due to the page limit set by the journal, these methodological details were only mentioned concisely in the introduction and are discussed comprehensively in the Materials and Methods section

12: The methodology section of the paper is confusing and lacks a clear summary. I suggest the authors include a detailed flowchart that outlines the sequence of steps taken in the study. This would greatly improve the readability and help readers understand the research process more quickly.

**Answer:**  We appreciate the reviewer’s valuable comment. In response, we have added a detailed flowchart (Figure 1) at the beginning of the *Materials and Methods* section to clearly illustrate the sequence of steps followed in this study. Furthermore, we have revised the opening paragraph of the section to provide a concise summary of the methodology before presenting each step in detail. These changes improve clarity and help readers better follow the research process.

13: When reporting a case study in a scientific paper, simply naming the very reliable vehicle (Tiba) equipment is not sufficient. You should provide detailed information such as the manufacturer, the factory, and the years of production. For example, in Iran, several companies manufacture similar equipment, so if it is not from Saipa, that should be clearly stated. All relevant details must be included to give full context. Moreover, the authors should explain why defect detection is particularly necessary for this type of equipment.

**Answer:** In the revised manuscript, we have provided comprehensive details regarding the case study. The analyzed component is used in the Tiba vehicle, manufactured by Saipa Automotive Company, and was produced by the Powder Metallurgy Factory in 2000. We have also elaborated on the importance of defect detection for this component. Even minor defects can lead to oil pump malfunction, which may subsequently cause insufficient engine lubrication, accelerated wear of moving parts, overheating, and ultimately, complete engine failure. These explanations have been incorporated into Section 2.4 (Experimental Setup and Data Acquisition) to enhance clarity and provide sufficient technical context.

14: How did the defects occur in the case studies? Were similar types of defects observed among the samples?

**Answer:**  The defects were identified through a structured inspection process conducted over a six-month period, during which cracked and defective components were systematically collected from the production line. Subsequent analysis showed that many of these samples shared similar types of flaws, particularly cracks along the internal surfaces of the stators. The recurrence of such defects indicates that they are not random in nature but are most likely associated with specific weaknesses or inconsistencies in the powder metallurgy manufacturing process. This repeated pattern emphasizes the importance of closely monitoring critical process parameters—such as compaction pressure, sintering temperature, and cooling rate—in order to reduce variability and enhance the overall reliability of the components. These clarifications have been integrated into Section 2.4 (Experimental Setup and Data Acquisition) to ensure greater transparency and technical detail.

15: The train-test splitting should not depend on only one specific random state. How can you generalize your model’s accuracy to other random states? I suggest using a “for” loop to change the random state multiple times and then report the mean values of all model metrics for both training and testing sets. Similar approaches were used in works such as “Explainable Artificial Intelligence Modeling of Internal Arc in a Medium Voltage Switchgear Based on Different CFD Simulations” and “Interpretable Machine Learning Modeling of Temperature Rise in a Medium Voltage Switchgear Using Multiphysics CFD Analysis.” In these studies, the mean metric values across different random states were reported, which improves the generalization of the models.

**Answer:** We sincerely thank you for raising this important point. In fact, the procedure you suggested was already implemented in our study, but due to an oversight and the routine nature of this approach, it was not clearly emphasized in the manuscript. Specifically, a 5-fold cross-validation method was applied, which involved 20 independent runs of the classifiers. In each run, approximately 80% of the samples in each class were randomly allocated for training, while the remaining 20% were used for testing. Following your valuable suggestion, we have revised the Materials and Methods section to explicitly describe the use of cross-validation and highlight its role in enhancing the generalizability of the models. Moreover, the relevant tables in the Results and Discussion section have been updated to report not only the average performance metrics of the classifiers but also their corresponding standard deviations across the different runs. We believe these clarifications and revisions directly address your concern and strengthen the transparency and robustness of the modeling framework presented in this study.

16: The formulation of the kernel function should be included in the paper. What about other kernel types? Also, did you use kernel functions with KNN? This method can achieve good results—for example, see “A Novel Machine Learning-Based Model for Predicting the Transition Fatigue Lifetime in Piston Aluminum Alloys.”

**Answer:**  We sincerely appreciate the reviewer’s insightful suggestion regarding the use of kernel functions. In this study, the KNN classifier was employed within a supervised learning framework, and all design parameters were systematically optimized through an extensive trial-and-error procedure. The configurations assessed included various nearest-neighbor search strategies (e.g., exhaustive, k-d tree), multiple distance metrics (e.g., Euclidean, Manhattan, Chebyshev, cosine, correlation, Mahalanobis, Minkowski), as well as different distance-weighting schemes (equal, inverse, squared inverse). The outcomes of these evaluations are comprehensively presented in the manuscript.

With respect to the reference suggested by the reviewer, we carefully reviewed the work and would like to highlight a key methodological distinction. The referenced study applied kernel functions within an unsupervised k-means clustering framework, whereas our research specifically implemented KNN as a supervised classification algorithm. Consequently, the design parameters pertinent to KNN were explicitly optimized and analyzed in the present study.

17: It is recommended to show the effect of training set size using figures and charts. For example, in “Modeling Nonlinear Deformation in Magnetic Polyelectrolyte Hydrogels: A Hybrid FEM-Machine Learning Framework,” there are figures titled “learning curves” that illustrate how the size of the dataset affects the model’s accuracy.

**Answer:**  We sincerely appreciate this valuable suggestion. Following your recommendation, Table 7 has been removed and replaced with Figure 8, which illustrates bar charts with standard deviations obtained through cross-validation. In addition, the corresponding description in Section 3.3 Impact of training set size on classifier generalization has been revised to reflect this modification.

18: For writing the conclusion, please present the main findings of the paper using bullet points. The conclusions should include both qualitative and quantitative results to clearly summarize the key outputs.

**Answer:**  We thank the reviewer for the valuable suggestion. In response, we have thoroughly revised the Conclusion section to clearly present the main findings using bullet points. Each bullet point now includes both qualitative and quantitative results, summarizing key outputs of the study in a concise and structured manner. The revised conclusion highlights the performance of all classifiers (SVM, KNN, MLP, and RBF) in terms of accuracy, specificity, robustness, and feature selection, as well as the impact of training set size, four-class classification results, and comparison with other NDT methods. Additionally, the section concludes with an overall statement on industrial relevance and a suggestion for future research.

**Reviewer # 2**

In this study, a defect detection method based on acoustic signal and machine learning is proposed, which has important engineering application value, reasonable research method, rigorous experimental design, and the results are innovative and practical. However, there is still room for improvement in the depth of the literature review, the completeness of the experimental details, the theoretical support of the analysis of the results, and the discussion of the limitations of the research. It is recommended that the author revise and improve the following issues:

1.“Page 3”

The introduction provides a comprehensive introduction to the existing non-destructive testing techniques, but there is little description of acoustic resonance testing (ART).

For powder metallurgy materials, the non-destructive testing technology used in the existing research is not introduced, and it is suggested to supplement and enhance the necessity and innovation of the research.

**Answer:** We appreciate the reviewer’s insightful comment. In Acoustic Resonance Testing (ART), excitation is applied to the component either mechanically or electromagnetically, and the vibrational response or resonance spectrum is precisely measured. Defects such as cracks, porosity, or internal flaws alter the natural frequencies and amplitudes of vibrations, which can be detected through acoustic signal analysis. This provides a non-destructive and accurate inspection technique, particularly suitable for parts manufactured by powder metallurgy.

We would like to inform the reviewer that the Introduction has been completely revised and restructured following the Editor’s guidance, emphasizing conciseness (limited to three pages) while including a thorough review of the available literature. We acknowledge that, in this specific domain, prior research is limited, which represents an inherent constraint of this study. To address this, Table 10 has been added in the Results section to summarize recent studies, and relevant references have been incorporated to compare and discuss their findings in relation to our work. We hope that these changes adequately address the reviewer’s concern, and we would greatly appreciate any additional suggestions to further improve this aspect

2.“Page 10”

The training process of machine learning models is not fully described in the experimental part, please supplement the specific process and standards of hyperparameter optimization of each model.

**Answer:**  We have revised the Materials and Methods section, particularly the subsections related to the classifiers, to the extent possible in order to address your comment. Additional details on the training process and the hyperparameter optimization procedures for each model have been provided wherever feasible.

3.“Page 13”

The excitation method is an electromagnetic exciter, but the magnitude of the excitation force, the position of the application point and the repeatability test results are not specified.

**Answer:** In response to your comment, additional details have been included in Section 2.4.1 *Equipment and Instrumentation* to clarify the excitation procedure. Specifically, we described that the parts were positioned 1 cm from the actuator tip, a constant excitation force of 10 N was applied, and the angular position of force application was selected randomly due to the geometric symmetry of the parts. We also highlighted that the procedure ensured consistent and reproducible acquisition of acoustic signals for subsequent analysis.

4.“Page 14”

Supplemental Fig. 3 for the longitudinal axis units.

**Answer:** We appreciate your valuable comment regarding the labeling of the axes. To improve clarity, the vertical axis in the time-domain plot has been revised to “Amplitude (Voltage)”, and in the frequency-domain plot to “Amplitude Spectrum”. In addition, the figure captions have been modified accordingly to ensure better understanding.

5.“Page 19”

The comparison of model performance (such as SVM, KNN, MLP, RBF) is comprehensive, but the analysis of the difference in detection difficulty of different defect types is insufficient, and the differences in the performance of models on different defect types and the reasons are not deeply discussed.

**Answer:**  We appreciate this valuable comment. Based on your suggestion, a discussion of the differences in detection difficulty among defect types has been added to the end of Section 3.2 *Overall Classifier Performance Evaluation*. In brief, although all classifiers achieved high overall accuracy, defects with strong acoustic signatures, such as severe cracks, were consistently detected by all models. Subtle defects, including micro-porosity and shallow surface flaws, were slightly more challenging, leading to occasional false positives for the SVM and MLP classifiers. This indicates that classifiers with stronger nonlinear mapping capabilities, such as KNN and RBF, are more effective in capturing subtle spectral variations. The differences in performance are likely due to the higher sensitivity of SVM and MLP to small overlaps in feature space, while KNN and RBF better preserve local neighborhood information and nonlinear separability.

6.“Page 26”

In the feature selection section, the optimal combination of features (e.g., PA3, S3, P4, C4, etc.) is determined, but the association of these features with the defect mechanism is not explained.

**Answer:**  We appreciate this comment. In response, a detailed explanation linking the selected features to the defect mechanisms has been added to the end of Section 3.4 *Feature Selection for Optimal Classification*. In brief, the optimal features (e.g., PA3, S3, P4, C4) were chosen based on their contribution to classification accuracy and their relevance to the physical defects in PM components. PA3 (Peak Amplitude of the third sub-band) reflects vibration intensity caused by cracks or fractures, with cracked components producing higher peaks at specific frequencies. S3 (Skewness of the third sub-band) indicates asymmetry in amplitude distribution, which is sensitive to irregularities such as chipping or tooth breakage. P4 (Peak Frequency of the fourth sub-band) corresponds to shifts in natural frequency caused by material loss or fractures, while C4 (Spectral Centroid of the fourth sub-band) represents the center of mass of the frequency spectrum, which shifts in the presence of structural defects. These features collectively capture both amplitude and frequency characteristics associated with different defect types, enabling accurate differentiation between intact and defective PM stators.

7.“Page 27”

Explain in detail how Fig. 5 distinguishes between intact and defective classification boundaries.

**Answer:** Figure 5 illustrates the classification boundaries between intact and defective PM stators based on the four selected features (P3, P4, S3, and MA4). As the classification space is inherently four-dimensional, it is not possible to create a fully representative visual for readers. To provide an interpretable two-dimensional view, each subplot was generated by varying two features within their ranges while keeping the remaining features fixed at their mean values. The colored regions indicate the decision zones defined by the classifier, with blue representing defective regions and brown indicating intact regions. Subplots (a)–(f) show all possible pairwise combinations of the four features, highlighting how the classifier separates the feature space into distinct regions. Blue clusters mark the locations of defective samples, whereas the brown background shows where intact samples dominate. The boundary shapes reflect the classifier’s ability to handle overlapping and nonlinear distributions. Overall, Figure 5 provides a visual approximation of the classifier’s decision boundaries and demonstrates how the selected features contribute to distinguishing intact and defective PM stators.

8.“Page 29”

The conclusions summarize the research results, but the limitations of the studies are not well discussed.

**Answer:**  Thank you for your comment. In response, a discussion addressing the study limitations has been added to the end of the Conclusions section. The added text reads as follows:

"In conclusion, combining acoustic resonance testing with machine learning classifiers offers a robust, efficient, and scalable approach for inspecting PM components. Although the method demonstrates high accuracy in defect detection, certain limitations remain. It does not provide precise information on defect location within the component, nor does it quantify the severity or size of detected anomalies. As a result, the extent of damage or changes in material properties cannot be assessed solely from the extracted features. These limitations may restrict the method’s application in industrial decision-making, where accurate information on defect location and severity is essential. Future work should focus on addressing these limitations by developing approaches for defect localization and severity estimation, implementing automated feature selection, enabling real-time analysis, and extending the methodology to other types of industrial machinery."

9.In the Results and Discussion section, it is recommended to cite the research results of others for discussion to support the research in this paper.

**Answer:** We appreciate the reviewer’s valuable suggestion. In response, the Results and Discussion section has been thoroughly revised and restructured. All discussions now include citations of relevant prior research to support and contextualize the findings of this study. This revision has been carried out in accordance with the recommendations of the reviewer, the Editor, and Reviewer 1, ensuring that the discussion is comprehensive, well-supported, and aligned with scientific writing standards.

----------------------------------------------------------------------------------------------------------------

We sincerely thank you for your insightful and constructive comments. Your valuable feedback has been instrumental in refining and improving the quality of our manuscript. We remain fully open to any additional suggestions you may have and are committed to incorporating all relevant recommendations in the final version of the paper.

**Abbas Rohani**

Department of Biosystems Engineering, Faculty of Agriculture, Ferdowsi University of Mashhad, Mashhad, Iran

ORCID: <https://orcid.org/0000-0002-4494-7058>

*Corresponding author: Abbas Rohani, Email:* [*arohani@um.ac.ir*](mailto:arohani@um.ac.ir)
