# Supplementary material for: Non-destructive defect detection in powder metallurgy automotive oil pump stators using acoustic signals and machine learning classification
Source: PLoS One. 2025 Nov 17;20(11):e0334454. doi: 10.1371/journal.pone.0334454 (PMC12622811; doi:10.1371/journal.pone.0334454)
Supplement: Attachment — Submitted filename: ReviewResponse19061404.docx [file pone.0334454.s002.docx]

**Manuscript Title:**

**Non-destructive defect detection in powder metallurgy components using acoustic signals and machine learning classification**

**Manuscript PONE-D-25-28300R2**

**Dear Editor in Chief,**

We would like to sincerely thank you and the reviewers for the time and effort devoted to evaluating our manuscript. The constructive comments and insightful suggestions provided during the first round of review were invaluable in improving the overall quality and clarity of the work.

We also wish to express our gratitude to the reviewers for their valuable contributions. At the same time, we would like to apologize for any difficulties encountered in tracking the extensive revisions made in the first round. Due to the large volume of modifications, the traceability of changes may not have been sufficiently clear.

In this revised submission, we have carefully addressed all remaining concerns. To ensure clarity and ease of review, the modifications have been clearly highlighted and page numbers have been indicated: changes made in response to the **Editor’s comments are highlighted in green**, while those addressing the **reviewers’ comments are highlighted in yellow**.

We hope that the manuscript in its current form meets the journal’s standards and satisfactorily addresses all comments

Abbas Rohani

**Additional Editor Comments:**

Besides considering the reviewer's comments, there are still several issues on the revised manuscript, as follows,

**1)** There are two equations for the number of (1).

**Answer:**  You are absolutely right, and the numbering of the formulas has been carefully reviewed and corrected. The revisions have been highlighted in green on **page 17**, within subsection “**2.6. Evaluation of classifier performance”.**

**2)** Almost all parts of the results section are yellow and it is confusing that either the discussion is improved or not.

**Answer:**  As you are aware, during the first round of revision the volume of required modifications was substantial. Among the major changes, the introduction had to be condensed to a maximum of three pages based on your recommendation, and the overall English standard of the manuscript also needed significant improvement. In addition, several extensive revisions were requested across other sections of the paper. These combined efforts unfortunately led to difficulties in clearly tracking and distinguishing the revised parts, which may have caused confusion.

We sincerely apologize for this issue. In the current round, special care has been taken to precisely address and clearly highlight the changes within the text so that they can be easily traced by both you and the reviewers

**3)** The discussion is to described the details and reasons of the obtained behavior, plus the comparison of the results to others in references.

**Answer:**  As was mentioned in the first round of revision, in accordance with your valuable suggestion and also your Comment No. 6 from the initial review, **Table 10** has been added on page 31 together with its explanation. In addition, the discussion has been further expanded and the results have been compared with those of other studies on **pages 29–30**. These modifications, which were made to strengthen the discussion and comparison of findings, have been highlighted in green for clarity. We hope that these revisions satisfactorily address your concern

**4)** It is not clear which reference is new! No yellow one!

**Answer:** We sincerely thank you for your kind remark. You are absolutely right, and this point was unfortunately overlooked in the first round of revision. In the current version, the newly added references have been clearly highlighted in green. Please kindly note that **references 31 to 53** are new additions to the reference list. These can be found on the **last three pages of the manuscript (pp. 36–38).**

**5)** The conclusions is lengthy and it should be shortened.

**Answer:** As noted in Comment No. 18 from Reviewer 1, the conclusion section was previously revised to present the main findings in bullet points, including both qualitative and quantitative results. However, in accordance with your valuable recommendation, this section has now been further shortened while still retaining the key outcomes of the study. We hope that the revised version addresses your concern appropriately **(pp. 32–33).**

**Reviewer # 1**

*The revised files are not acceptable in their current form. The authors should clearly highlight only the exact changes made in the manuscript rather than highlighting entire sections. At present, large portions of the text are highlighted in yellow, even where no changes appear to have been made. This makes it very difficult to evaluate whether the authors have addressed my comments effectively.*

I strongly request that:

1:The authors highlight only the specific text that was changed or added, not entire sections.

2:The response-to-reviewers document should clearly list and describe all changes made in the manuscript, including page and line numbers where applicable.

3:The highlighting should be revised to accurately reflect changes to ensure clarity during review.

With the current formatting, it is not possible to determine whether my previous comments have been adequately addressed.

**Answer:** We fully agree with your observation and sincerely apologize for the difficulties caused. In the first round of revision, the extensive changes requested by the *Editor*—including condensing the introduction to a maximum of three pages, revising the conclusions and other sections, and improving the manuscript’s English—resulted in large portions of the text being highlighted in yellow.

We acknowledge that this made it difficult to track the specific changes corresponding to your comments. In the current revision, only the modifications related to your comments are highlighted in yellow, while changes requested by the Editor are highlighted in green. We also note that the changes suggested by *Reviewer #2*, which were already addressed in the first round, were not highlighted previously.

Finally, all of your comments have been carefully addressed again in the response-to-reviewers document, with precise page and line references provided to ensure clarity and traceability of each modification.

We hope that these revisions now meet your expectations

I recommend the paper for major revision with the following comments:

**1:** The title of the paper is too general, especially since the authors mention “powder metallurgy components” without specifying the material. This approach overgeneralizes the results obtained from a limited number of samples to the entire manufacturing process.

**Answer:**  Thank you for your valuable comment. We agree with your observation that the previous title was too general. To address this concern and to more accurately reflect the specific scope of our study, we have revised the title to:

*“Non-destructive defect detection in powder metallurgy automotive oil pump stators using acoustic signals and machine learning classification.”*

This revision avoids overgeneralization and specifies the exact type of component investigated, ensuring that the results are clearly contextualized within automotive oil pump stators manufactured by powder metallurgy.

*The change has been made on page 1 of the manuscript.*

**2:** You should avoid using overly polished or complicated text in a research paper. For example, the sentence “Furthermore, PM manufacturing typically involves high production volumes, necessitating not only accurate defect detection but also rapid inspection methods” is unnecessarily complex. It uses correlative conjunctions within a present participle clause, which makes it difficult to follow. Also, avoid combining phrases like “due to” and “however” in one sentence. It would be clearer to restructure the sentence using a single transition word such as “although.” Initially, I mentioned this issue in the abstract, but after reviewing the rest of the paper, I recommend checking the entire text. Although the language is grammatically correct, it is sometimes hard to read. A research paper should use clear, direct, and consistent structures — it should not include a variety of sentence forms like TOEFL or IELTS essays.

**Answer:**  We have carefully reviewed the entire manuscript and revised the text wherever possible to simplify complex sentences, improve clarity, and ensure direct and consistent expression throughout the paper. *In particular, the introduction was rewritten and condensed according to the Editor’s recommendation to limit it to three pages*, which involved removing or summarizing many sentences. Moreover, the entire manuscript was reviewed again based on your suggestion, and numerous improvements were made to enhance readability and clarity. We hope these revisions address your concerns satisfactorily.

Specifically, the sentence you highlighted in the abstract has been rewritten and modified (see *page 1, line 5*), and we note that the remainder of the abstract has also been revised in accordance with the recommendations of the Editor and Reviewer #2.

**3:** The advantage of using machine learning (ML) in this research is not mentioned in the abstract. It should be explained how this method can be effective and how it compares with other conventional methods. Currently, the abstract only highlights the necessity of using acoustic signal testing, without emphasizing the role or benefits of ML. Additionally, instead of providing a long general introduction in the abstract, it would be more useful to include details about the algorithm training process. Overall, the abstract needs to be completely restructured.

**Answer:**  We sincerely thank the Reviewer for this valuable comment. In the revised version, the Abstract has been improved to explicitly highlight the advantages of machine learning over conventional threshold-based inspection techniques, emphasizing its ability to detect complex, non-linear acoustic signal patterns that are often missed by traditional approaches. In addition, the training process of the algorithms has been clarified by specifying that time- and frequency-domain features were extracted from the collected acoustic data and used to train and validate four supervised ML classifiers (SVM, k-NN, MLP, and RBF). Furthermore, the comparative results of these models have been briefly summarized to demonstrate their performance, with the RBF model achieving 100% accuracy.

*The changes can be found in the Abstract on page 1 of the manuscript.*

**4:** How is it possible that SVM and MLP neural network are listed as keywords in your paper but are not mentioned in the title or abstract? These important terms should at least appear in the abstract to reflect the main methods used in the study.

**Answer:**  We appreciate the Reviewer’s insightful observation. In accordance with your suggestion and the Editor’s guidance, the list of keywords has been carefully revised, and the Abstract has been rewritten to ensure that all major methods, including SVM and MLP, are explicitly mentioned. This issue has been fully addressed in the revised manuscript, and the updated list of keywords can be reviewed for confirmation

*The changes can be found in the Keywords section on page 1 of the manuscript.*

5: Please avoid introducing abbreviations for the first time without proper definition. When using an abbreviation, it should be introduced once with its full form, and then only the abbreviation should be used throughout the paper. For example, you have introduced “PM” and “ML” several times in different sections. Please check and correct this issue for all abbreviations in the text.

**Answer:**  We sincerely thank the Reviewer for this valuable comment. Following your suggestion, we have carefully revised the manuscript to ensure that each abbreviation is introduced only once with its full form at the first appearance, and then consistently used throughout the text. Specifically, abbreviations such as “PM” and “ML” have been corrected accordingly. This revision has been applied uniformly across all sections of the manuscript.

*The corrections are reflected throughout the manuscript.*

**6:** In the introduction, several long sentences make it difficult to follow the ideas clearly. The sentence structures are similar to those typically found in TOEFL reading passages, which are unnecessarily complex for a research paper. I recommend simplifying and restructuring these sentences to improve clarity and readability.

**Answer:**  We appreciate the reviewer’s valuable comment. Following the editor’s guidance to reduce the introduction to a maximum of three pages, this section has been revised accordingly. In addition, the issue rightly noted by the reviewer regarding long and complex sentence structures has been carefully addressed, not only in the introduction but also throughout other sections of the manuscript to enhance clarity and readability.

*Since the entire introduction was extensively rewritten, all the points you highlighted have been incorporated.* ***Please refer to pages 3–5 of the manuscript to review these changes.***

**7:** The introduction is too long, and the ideas in the paragraphs are not well connected. As a result, it is difficult for the reader to follow the overall direction of the paper. Moreover, the extended explanation about different types of non-destructive inspection methods makes the introduction unnecessarily lengthy and somewhat boring. In particular, the second paragraph does not contribute to the main objective of the paper. Instead, it seems to list information without offering a clear link to the research focus. Therefore, I suggest reorganizing and shortening the introduction to improve clarity, relevance, and reader engagement.

**Answer:**  We sincerely appreciate your valuable feedback. In line with your recommendation and the editor’s suggestion, the introduction has been thoroughly revised and reorganized. The section was shortened to improve clarity, eliminate redundancies, and strengthen the logical flow of ideas. The part concerning non-destructive inspection methods was also streamlined to ensure relevance to the research objectives. We hope that these revisions adequately address your concern.

*Based on both your comment and the Editor’s guidance, the introduction was reduced to three pages and substantially rewritten; therefore, we did not highlight the entire section.* ***However, the last two paragraphs of the introduction were specifically revised in response to this comment and highlighted in yellow for your convenience (pages 5).*** *Please refer to pages 3–5 of the manuscript to review these changes.*

**8:** The long explanation of vibration non-destructive testing in paragraph 3 of the introduction may be boring for the reader. It would be better to shorten this section and focus more on information directly relevant to the study.

**Answer:**  We appreciate your observation. This section has been shortened and simplified to avoid unnecessary detail. The primary aim of including it was to introduce the subject and highlight the importance of applying machine learning methods in this research area. In line with your valuable feedback, we have revised and restructured this part to improve clarity and relevance.

**9:** The literature review in the introduction related to acoustic testing is insufficient. Instead of listing different types of quality control methods and inspections in paragraph 2 and providing a long introduction to vibration testing at the beginning of paragraph 3, please include more detailed information about how acoustic testing differs from other methods. Also, explain the necessity of using this method compared to others, recent developments in acoustic testing, and its advantages.

**Answer:** We appreciate your comment regarding the coverage of acoustic testing in the introduction. The introduction has been thoroughly revised and streamlined to avoid unnecessary elaboration. However, based on our review and the available references, there is very limited research on defect detection in industrial components produced by powder metallurgy, particularly in the automotive sector. Therefore, to highlight the importance of acoustic testing, we referred to related studies where applicable, in order to clarify its relevance and advantages compared to other methods. Additionally, Table 10 (*Page 30*) has been added to the manuscript, providing a comprehensive overview of the literature in this area. As shown in the table, most existing studies focus on defect detection using image-based techniques, which further emphasizes the novelty and significance of our work in applying acoustic testing for industrial defect detection.

**10:** Before the last paragraph of the introduction, which presents the novelty of the paper, the authors should clearly elaborate on the research gap and the necessity of conducting this study. Specifically, the following questions need to be addressed in detail:

- What are the necessities of using machine learning classification in this context?

- What advantages does the proposed method offer over existing approaches?

I recently reviewed a paper for PLOS One that used classification for defect detection in aluminum samples. I am interested to know whether changing the materials, manufacturing methods, or machine learning algorithms can contribute to solving real-world problems. If this method provides new insights or tools for researchers, the authors should explain this clearly in the research gap section of their paper.

**Answer:** We appreciate the reviewer’s valuable comment. In accordance with your suggestion, the necessity of using machine learning classification in this context and the advantages of the proposed method compared to existing approaches have been clarified at the end of the introduction. In particular, the study by Han et al. [Han, Ying, Xingkun Li, Gongxiang Cui, Jie Song, Fengyu Zhou, and Yugang Wang. Multi-defect detection and classification for aluminum alloys with enhanced YOLOv8. PloS One, 20(3), e0316817, 2025] was reviewed. As also highlighted in our introduction, nearly all of these studies are image-based, which suffer from certain drawbacks such as being time-consuming, costly, and lacking sufficient speed for continuous production lines. These limitations provided the main motivation for our research, where we propose a machine learning approach based on acoustic signals that enables faster, cost-effective, and more practical defect detection for real-world applications.

**11:** At the end of the introduction, the authors should provide the reader with a clear summary of the novelty and methodology. This should include how the samples were prepared, how the algorithms were trained, and whether different algorithms were compared to find the best one. It should also explain how the accuracy of the models was evaluated, if train-test splitting was used to assess the ability to predict new samples, and what materials were tested. All these details should be clearly defined in the last paragraph of the introduction.

**Answer:**  In the final paragraph of the introduction, we highlighted the research gap and the novelty of the study, while briefly outlining the main steps of the methodology, including sample preparation, algorithm training, and model evaluation. However, due to the page limit set by the journal, these methodological details were only mentioned concisely in the introduction and are discussed comprehensively in the Materials and Methods section. If there are still any specific points that require further clarification, we would be grateful if you could kindly indicate them so that we can address them thoroughly.

**12:** The methodology section of the paper is confusing and lacks a clear summary. I suggest the authors include a detailed flowchart that outlines the sequence of steps taken in the study. This would greatly improve the readability and help readers understand the research process more quickly.

**Answer:** We sincerely thank the Reviewer for this constructive suggestion. In response, a detailed flowchart (Figure 1) has been added at the beginning of the *Materials and Methods* section (*end of page 5 and page 6*) to illustrate the sequence of steps carried out in this study. In addition, the opening paragraph of the section has been revised to provide a concise summary of the methodology before each step is described in detail. These revisions significantly improve clarity and allow readers to follow the research process more easily.

**13:** When reporting a case study in a scientific paper, simply naming the very reliable vehicle (Tiba) equipment is not sufficient. You should provide detailed information such as the manufacturer, the factory, and the years of production. For example, in Iran, several companies manufacture similar equipment, so if it is not from Saipa, that should be clearly stated. All relevant details must be included to give full context. Moreover, the authors should explain why defect detection is particularly necessary for this type of equipment.

**Answer:** In the revised manuscript, we have provided comprehensive details regarding the case study. The analyzed component is used in the Tiba vehicle, manufactured by Saipa Automotive Company, and was produced by the Powder Metallurgy Factory in 2000. We have also elaborated on the importance of defect detection for this component. Even minor defects can lead to oil pump malfunction, which may subsequently cause insufficient engine lubrication, accelerated wear of moving parts, overheating, and ultimately, complete engine failure. These explanations have been incorporated into Section 2.4 (Experimental Setup and Data Acquisition) to enhance clarity and provide sufficient technical context (*page 10*).

**14:** How did the defects occur in the case studies? Were similar types of defects observed among the samples?

**Answer:** The defects were identified through a structured inspection process conducted over a six-month period, during which cracked and defective components were systematically collected from the production line. Subsequent analysis showed that many of these samples shared similar types of flaws, particularly cracks along the internal surfaces of the stators. The recurrence of such defects indicates that they are not random in nature but are most likely associated with specific weaknesses or inconsistencies in the powder metallurgy manufacturing process. This repeated pattern emphasizes the importance of closely monitoring critical process parameters—such as compaction pressure, sintering temperature, and cooling rate—in order to reduce variability and enhance the overall reliability of the components. These clarifications have been integrated into Section 2.4 (Experimental Setup and Data Acquisition) to ensure greater transparency and technical detail. (*page 10*).

**15:** The train-test splitting should not depend on only one specific random state. How can you generalize your model’s accuracy to other random states? I suggest using a “for” loop to change the random state multiple times and then report the mean values of all model metrics for both training and testing sets. Similar approaches were used in works such as “Explainable Artificial Intelligence Modeling of Internal Arc in a Medium Voltage Switchgear Based on Different CFD Simulations” and “Interpretable Machine Learning Modeling of Temperature Rise in a Medium Voltage Switchgear Using Multiphysics CFD Analysis.” In these studies, the mean metric values across different random states were reported, which improves the generalization of the models.

**Answer:** We sincerely thank you for raising this important point. In fact, the procedure you suggested was already implemented in our study, but due to an oversight and the routine nature of this approach, it was not clearly emphasized in the manuscript. Specifically, a 5-fold cross-validation method was applied, which involved 20 independent runs of the classifiers. In each run, approximately 80% of the samples in each class were randomly allocated for training, while the remaining 20% were used for testing. Following your valuable suggestion, we have revised the Materials and Methods section to explicitly describe the use of cross-validation and highlight its role in enhancing the generalizability of the models. Moreover, the relevant tables in the Results and Discussion section have been updated to report not only the average performance metrics of the classifiers but also their corresponding standard deviations across the different runs. We believe these clarifications and revisions directly address your concern and strengthen the transparency and robustness of the modeling framework presented in this study.

**The applied changes can be reviewed at the following locations:**

1. *Materials and Methods*, Section 2.6, *Evaluation of classifier performance*, start and end of page 17
2. Table 1 and its description, page 19
3. Table 2 and its description, page 20
4. Table 3 and its description
5. Table 4 and its description
6. Figure 6 and its description
7. Table 8 and its description
8. Table 9 and its description

**16:** The formulation of the kernel function should be included in the paper. What about other kernel types? Also, did you use kernel functions with KNN? This method can achieve good results—for example, see “A Novel Machine Learning-Based Model for Predicting the Transition Fatigue Lifetime in Piston Aluminum Alloys.”

**Answer:**  We sincerely appreciate the reviewer’s insightful suggestion regarding the use of kernel functions. In this study, the KNN classifier was employed within a supervised learning framework, and all design parameters were systematically optimized through an extensive trial-and-error procedure. The configurations assessed included various nearest-neighbor search strategies (e.g., exhaustive, k-d tree), multiple distance metrics (e.g., Euclidean, Manhattan, Chebyshev, cosine, correlation, Mahalanobis, Minkowski), as well as different distance-weighting schemes (equal, inverse, squared inverse). The outcomes of these evaluations are comprehensively presented in the manuscript.

With respect to the reference suggested by the reviewer, we carefully reviewed the work and would like to highlight a key methodological distinction. The referenced study applied kernel functions within an unsupervised k-means clustering framework, whereas our research specifically implemented KNN as a supervised classification algorithm. Consequently, the design parameters pertinent to KNN were explicitly optimized and analyzed in the present study.

**17**: It is recommended to show the effect of training set size using figures and charts. For example, in “Modeling Nonlinear Deformation in Magnetic Polyelectrolyte Hydrogels: A Hybrid FEM-Machine Learning Framework,” there are figures titled “learning curves” that illustrate how the size of the dataset affects the model’s accuracy.

**Answer:**  We sincerely appreciate this valuable suggestion. Following your recommendation, Table 7 has been removed and replaced with Figure 8 (*Page 24*), which illustrates bar charts with standard deviations obtained through cross-validation. In addition, the corresponding description *in Section 3.3* Impact of training set size on classifier generalization has been revised to reflect this modification.

**18:** For writing the conclusion, please present the main findings of the paper using bullet points. The conclusions should include both qualitative and quantitative results to clearly summarize the key outputs.

**Answer:**  We thank the reviewer for the valuable suggestion. In response, we have thoroughly revised the Conclusion section to clearly present the main findings using bullet points. Each bullet point now includes both qualitative and quantitative results, summarizing key outputs of the study in a concise and structured manner. The revised conclusion highlights the performance of all classifiers (SVM, KNN, MLP, and RBF) in terms of accuracy, specificity, robustness, and feature selection, as well as the impact of training set size, four-class classification results, and comparison with other NDT methods. Additionally, the section concludes with an overall statement on industrial relevance and a suggestion for future research *(Page 32).*

**Reviewer # 2**

The paper is now significantly improved and I am happy to recommend publication. Manuscript improved quality. It is acceptable.

**Answer:**  We sincerely appreciate your valuable comments and constructive feedback, which have greatly contributed to improving the quality of our manuscript. We also apologize for any inconvenience caused by the insufficient traceability of the revisions in the earlier round. Your support and recommendation for publication are highly appreciated.

----------------------------------------------------------------------------------------------------------------

We sincerely thank you for your insightful and constructive comments. Your valuable feedback has been instrumental in refining and improving the quality of our manuscript. We remain fully open to any additional suggestions you may have and are committed to incorporating all relevant recommendations in the final version of the paper.

**Abbas Rohani**

Department of Biosystems Engineering, Faculty of Agriculture, Ferdowsi University of Mashhad, Mashhad, Iran

ORCID: <https://orcid.org/0000-0002-4494-7058>

*Corresponding author: Abbas Rohani, Email:* [*arohani@um.ac.ir*](mailto:arohani@um.ac.ir)
