# Supplementary material for: Non-destructive defect detection in powder metallurgy automotive oil pump stators using acoustic signals and machine learning classification
Source: PLoS One. 2025 Nov 17;20(11):e0334454. doi: 10.1371/journal.pone.0334454 (PMC12622811; doi:10.1371/journal.pone.0334454)
Supplement: Attachment — Submitted filename: ReviewResponse24061404.docx [file pone.0334454.s003.docx]

**Manuscript Title:**

**Non-destructive defect detection in powder metallurgy components using acoustic signals and machine learning classification**

**Manuscript PONE-D-25-28300R3**

**Dear Editor in Chief,**

We sincerely thank you and the reviewers for the time and effort devoted to evaluating our manuscript. The constructive comments and insightful suggestions provided during the review process were invaluable in improving the quality, clarity, and overall presentation of our work.

We also wish to express our gratitude to the reviewers for their detailed and professional feedback. At the same time, we apologize for any difficulties encountered in tracking the extensive revisions made in the second round. Due to the substantial number of modifications, some changes may not have been immediately evident, and we appreciate your understanding in this regard.

We believe that the manuscript, in its current revised form, fully addresses the reviewers’ comments and meets the standards of *PLOS ONE*. We hope it will now be considered suitable for publication.

**Additional Editor Comments:**

**1)** The conclusions section is still lengthy.

**Answer:** The Conclusions section has been further revised and shortened to ensure conciseness as requested.

" **Conclusions**

This study demonstrated that acoustic resonance testing combined with ML classifiers can accurately detect defects in powder metallurgy components. The main findings are summarized as follows:

- SVM achieved 99% accuracy, KNN reached 100% accuracy, and both MLP and RBF classifiers showed near-perfect training and testing performance. These results demonstrate the robustness and reliability of the models.
- KNN and RBF achieved perfect overall performance, while SVM and MLP reached approximately 0.99 accuracy with minor false positives. RBF maintained near-perfect generalization using only 50% of the training data. In contrast, SVM and MLP were more sensitive to smaller datasets, and KNN showed slight decreases in testing accuracy.
- Carefully chosen RBF features from the third and fourth frequency ranges enabled perfect classification, including four-class distinction. This shows that dimensionality can be reduced without losing robustness and highlights the importance of mid-to-high frequency features for distinguishing defect types.
- Acoustic resonance testing combined with ML achieved 100% accuracy, outperforming other non-destructive methods. This approach enables reliable quality control and fault diagnosis of PM components using 10–20 kHz frequency bands.

In conclusion, combining acoustic resonance testing with ML provides a robust, efficient, and scalable approach for inspecting PM components. Although the method is highly accurate, it does not provide information on defect location or severity, which limits its applicability in industrial decision-making. Future work should focus on developing methods for defect localization and severity estimation, implementing automated feature selection, enabling real-time analysis, and extending the approach to other industrial components."

-----------------------------------------------------------------------------------------------------------------

**Reviewer # 1**

*I cannot reject this paper, but I also cannot accept it. Therefore, I recommend that additional reviewers carefully evaluate the manuscript in this major revision, but I must emphasize that this will be the final review round. I will not accept further revisions beyond this because I am doubtful about the accuracy of this paper. While the methodology and overall work are promising, the authors have been inaccurate in addressing even minor comments. This raises concerns about the reliability of the presented results and representations. I am doubtful that other, more significant issues may exist that I have not identified.*

**Answer:**  We sincerely thank the reviewer for the insightful and constructive feedback, which has substantially improved the quality and clarity of this manuscript.

Because the first revision required extensive rewriting, many sections were fully restructured.
In the second round, highlights were limited to the unresolved points to avoid confusion, which may have made some changes less visible.

We apologize for any inconvenience and summarize below the key revisions implemented.

***Overview of major revisions implemented in response to the constructive comments and recommendations provided during both the first and second rounds of review.***

1. ***Title Refinement*** *– Revised to “Non-destructive defect detection in powder metallurgy automotive oil pump stators using acoustic signals and machine learning classification” to ensure specificity and avoid overgeneralization.*
2. ***Language and Style*** *– Entire manuscript carefully edited to shorten sentences, improve readability, and maintain a clear, direct scientific tone.*
3. ***Abstract Enhancement*** *– Completely rewritten to emphasize the advantages of machine learning, include key training details, and report the performance of the four classifiers (SVM, k-NN, MLP, RBF). Keywords updated accordingly.*
4. ***Introduction*** *– Reduced to three pages, reorganized for logical flow, and revised to clearly present the research gap, motivation for ML, and novelty while removing redundant NDT descriptions.*
5. ***Methodology*** *– Added a detailed flowchart (new Figure 1) summarizing experimental and modeling steps; opening paragraph rewritten for clarity.*
6. ***Case Study and Defects*** *– Provided full context on the Tiba vehicle, production site, defect types, and sample collection procedures.*
7. ***Modeling Details*** *– Expanded explanation of KNN parameters, kernel settings, and validation strategy (5-fold cross-validation with 20 runs); tables and figures updated with mean values and standard deviations.*
8. ***Results Presentation*** *– Replaced Table 7 with a new Figure 6 showing training-set size effects on accuracy with standard deviations.*
9. ***Conclusions*** *– Shortened and reformatted into concise bullet points summarizing key quantitative and qualitative findings, industrial implications, and directions for future research.*

***-****---------------------------------------------------------------------------------------------------------------*

***Several examples of minor issues include:***

**1.** Comment 17: The authors responded that “Table 7 has been removed and replaced with Figure 8.” However, Figure 8 does not exist in the revised manuscript.

**Answer:**  We appreciate the reviewer’s careful examination. It appears there was a mislabeling in our previous response. In the revised manuscript, **Figure 6**, not Figure 8, has replaced Table 7. Please see the top of **page 25**, where Figure 6 presents the corresponding results. We apologize for the confusion and thank the reviewer for noting this oversight.

**Figure 6.** Impact of training set size on the mean and standard deviation of SVM, KNN, MLP, and RBF classification accuracy under cross-validation

**2**. Abbreviations: Inconsistencies remain for terms such as “machine learning” (sometimes written in full, sometimes abbreviated), “accuracy” (defined as Acc but inconsistently used), and NN. Despite the authors’ claim that abbreviations were corrected, the manuscript still shows these inconsistencies.

**Answer:**  We sincerely thank the reviewer for pointing out this important issue. The entire manuscript has been thoroughly re-checked, and all abbreviations, including *machine learning (ML)*, *accuracy (Acc)*, and *NN*, have now been consistently revised throughout the text to ensure uniform usage.

- **Machine learning (ML):** Introduced in full at first mention and subsequently abbreviated as *ML* on pages 1, 3, 4, 5, 6, 7, 10, 29, 30, 31, 32, and 33.
- **Accuracy (Acc):** Used in full throughout the text, except in Table 6 (page 23), where it is presented as *Acc* together with other classifier evaluation metrics (e.g., TP, FP, Acc, Prec, Rec, F1, Spec, AUC, YI) due to space limitations. A note beneath the table clearly defines the abbreviation to maintain clarity.
- **NN:** The inconsistency in Table 10 has been corrected to ensure proper and consistent usage.

**3**. Highlighting changes: I requested that revisions be highlighted. This was not done. For example, in the abstract, a sentence about acoustic signals from different components was revised but not highlighted.

**Answer:** We sincerely apologize for the inconvenience caused. As requested by the editor and reviewers, the entire abstract was extensively rewritten to incorporate all comments and suggestions. Because the revisions involved a complete rephrasing rather than isolated sentence changes, it was not possible to highlight each specific modification individually, which may have made it difficult to track the changes. We kindly ask the reviewer to review the abstract again with this in mind, and we once again apologize for any difficulty this may have created.

**4.** Comment 16: My suggestion was to include the formulation of kernel functions, which is standard in scientific papers. Instead, the authors focused on a comparison between supervised and unsupervised machine learning, which was not requested.

**Answer:** We sincerely thank the reviewer for the valuable suggestion. In accordance with your recommendation, the following content has been added to the *Materials and Methods* section (**pages 14–15**) to include the formulations of the kernel functions:

" To handle nonlinear relationships, four kernel functions were evaluated: linear, polynomial of degree 2, polynomial of degree 3, and the RBF. These kernels allow the input features to be mapped into higher-dimensional spaces, improving class separability. Their mathematical formulations are presented in Eqs. (2)–(5):

$$K\left( x_{i},x_{j} \right)=x_{i}^{T}x_{j}$$

(2)

$$K\left( x_{i},x_{j} \right)={({\gamma x}_{i}^{T}x_{j}+r)}^{2}$$

(3)

$$K\left( x_{i},x_{j} \right)={({\gamma x}_{i}^{T}x_{j}+r)}^{3}$$

(4)

$$K\left( x_{i},x_{j} \right)=exp(-\gamma\left\| x_{i}-x_{j} \right\|^{2})$$

(5)

where *x*_i_ and *x*_j_ represent input feature vectors, γ>0 denotes the kernel scale parameter that controls the influence of individual samples, and rrr is the coefficient used in the polynomial kernels. These kernel functions enable the SVM classifier to construct both linear and highly nonlinear decision boundaries, thereby improving classification performance in high-dimensional datasets."

**Reviewer # 2**

The paper is now significantly improved and I am happy to recommend publication. Manuscript improved quality. It is acceptable.

**Answer:**  We sincerely appreciate your valuable comments and constructive feedback, which have greatly contributed to improving the quality of our manuscript. We also apologize for any inconvenience caused by the insufficient traceability of the revisions in the earlier round. Your support and recommendation for publication are highly appreciated.

----------------------------------------------------------------------------------------------------------------

We sincerely thank you for your insightful and constructive comments. Your valuable feedback has been instrumental in refining and improving the quality of our manuscript. We remain fully open to any additional suggestions you may have and are committed to incorporating all relevant recommendations in the final version of the paper.

**Abbas Rohani**

Department of Biosystems Engineering, Faculty of Agriculture, Ferdowsi University of Mashhad, Mashhad, Iran

ORCID: <https://orcid.org/0000-0002-4494-7058>

*Corresponding author: Abbas Rohani, Email:* [*arohani@um.ac.ir*](mailto:arohani@um.ac.ir)
